# Ruptured sinus of Valsalva aneurysm coexisted with congenital ventricular septal defect: a case series

**DOI:** 10.1186/s43044-023-00420-y

**Published:** 2023-11-06

**Authors:** Ni Made Ayu Wulan Sari, Made Satria Yudha Dewangga, Ida Bagus Rangga Wibhuti, Luh Oliva Saraswati Suastika, I Dewa Gede Surya Mahardika Badung, Bryan Gervais de Liyis, Anastasya Maria Kosasih, Gusti Ngurah Prana Jagannatha

**Affiliations:** 1 Department of Cardiology and Vascular Medicine, Prof. Dr. I.G.N.G Ngoerah General Hospital, Diponogoro street, Denpasar, Bali, 80113 Indonesia; 2 Department of Cardiology and Vascular Medicine, Faculty of Medicine, Udayana University/Prof. Dr. I.G.N.G Ngoerah General HospitalUdayana University, Udayana University Hospital, Denpasar, Bali, Indonesia; 3 Faculty of Medicine, Udayana University/Prof. Dr. I.G.N.G Ngoerah General Hospital, Denpasar, Bali, Indonesia

**Keywords:** Echocardiography, Rupture, Sinus of Valsalva aneurysm, Ventricular septal defect

## Abstract

**Background:**

Ruptured sinus of Valsalva aneurysm (RSoVA) is an uncommon cardiac anomaly that may occasionally coexist with additional congenital heart disease (CHD). The presence of such congenital cardiac anomalies, frequently involving a ventricular septal defect (VSD), is of significant clinical importance and warrants vigilant detection during echocardiographic assessments to prevent oversight.

**Case presentation:**

Three cases of RSoVA accompanied by VSD are presented in which all patients manifested symptoms of shortness of breath (SOB). In the first patient, right and left heart catheterization was undertaken; however, the images failed to reveal any evidence of VSD flow due to the occlusion of a small VSD by the prolapsed right coronary cusp (RCC). Prior to surgical intervention, multimodal imaging was conducted, revealing the presence of RSoVA extending into the right ventricle (RV) along with the VSD. The second patient had a prior childhood diagnosis of VSD but had not undergone further medical intervention. Transoesophageal echocardiography (TEE) was subsequently performed, identifying the presence of RSoVA, a small VSD, and valvular heart disease. The third patient presented with heart failure, exacerbated during her second pregnancy. TEE was also administered to this patient, revealing the presence of RSoVA accompanied by a small subaortic VSD with a left-to-right shunt. All three patients were scheduled for surgical repair of the ruptured sinus of Valsalva and closure of the VSD.

**Conclusions:**

The coexistence of RSoVA and CHD, typically VSD, is frequently observed in patients experiencing symptoms of SOB. Recognizing the presence of CHD in individuals with RSoVA is of paramount importance, as it can significantly influence their medical management and treatment strategies.

## Background

Sinus of Valsalva aneurysm (SoVA) is an abnormal dilatation of the aortic root located between the aortic valve annulus and the sinotubular junction that occurs at the elastic lamina [1]. SoVA is typically a congenital anomaly of rare occurrence. The prevalence of SoVA in the Asian population is around 1.2–1.8%, and approximately 0.14–0.96% in the Western population with congenital heart disease (CHD). This condition predominantly manifests in middle-aged males as opposed to females and is frequently found in association with Ventricular Septal Defects (VSD) [2]. Remarkably, SoVA often goes undetected during routine echocardiographic examinations. The enlargement of an aneurysm can lead to structural impairment, thereby causing symptoms related to the involvement of adjacent anatomical structures. Notably, SoVA most frequently originates from the right coronary cusp (RCC) at a prevalence rate ranging from 70 to 90%, followed by the non-coronary cusp (NCC) at 10% to 25%, and least commonly from the left coronary cusp (LCC) at less than 5% [3].

During the rupture of sinus Valsalva aneurysm (RSoVA), the blood flow from the rupture tends to drain into the right ventricle (RV), followed by the right atrium (RA). In certain instances, RSoVA can exhibit extensions into multiple cardiac chambers concurrently or can have drainage pathways into atypical locations, including the left ventricle (LV), left atrium (LA), interventricular septum, interatrial septum, superior vena cava, pulmonary artery, or even extending into the pericardial or pleural cavities. The clinical presentation of RSoVA displays a spectrum of variability, spanning from asymptomatic cases to symptomatic individuals experiencing heart failure, and in severe instances, culminating in fatality. The diagnostic process for RSoVA is greatly facilitated by employing multimodal cardiac imaging techniques, which serve both for definitive diagnosis and as a guide for interventional procedures. Echocardiography, often used alone or in conjunction with angiography, proves to be the primary modality for diagnosing most cases of SoVA [4]. The utilization of multimodality cardiac imaging plays a pivotal role in assisting physicians in the accurate diagnosis of RSoVA and the concurrent presence of CHD, notably VSD. In this context, we present a report detailing the clinical profiles of three patients who exhibited RSoVA with drainage into the RV concomitant with VSD.

## Case presentation

### Case I

A 28-year-old female presented at Prof. Dr. IGNG Ngoerah General Hospital with a chief complaint of dyspnea on exertion and bilateral leg swelling, persisting for the preceding three months leading up to her hospital visit. The patient reported no history of chronic illnesses, regular medication usage, or a smoking habit. Upon physical examination, several notable findings were identified, including an elevated jugular venous pressure, a continuous murmur graded as V/VI detected at the lower left sternal border, hepatomegaly, and bilateral leg edema. An electrocardiogram (ECG) displayed sinus tachycardia featuring a right axis deviation, right ventricular hypertrophy, and the presence of ventricular extrasystoles. Subsequently, a chest X-ray unveiled cardiomegaly, encompassing dilatation of all cardiac chambers, in conjunction with heightened pulmonary vascularization. Further evaluation via transthoracic echocardiography (TTE) revealed the dilation of all cardiac chambers, with concurrent preservation of left ventricular systolic function.

**Fig. 1:**
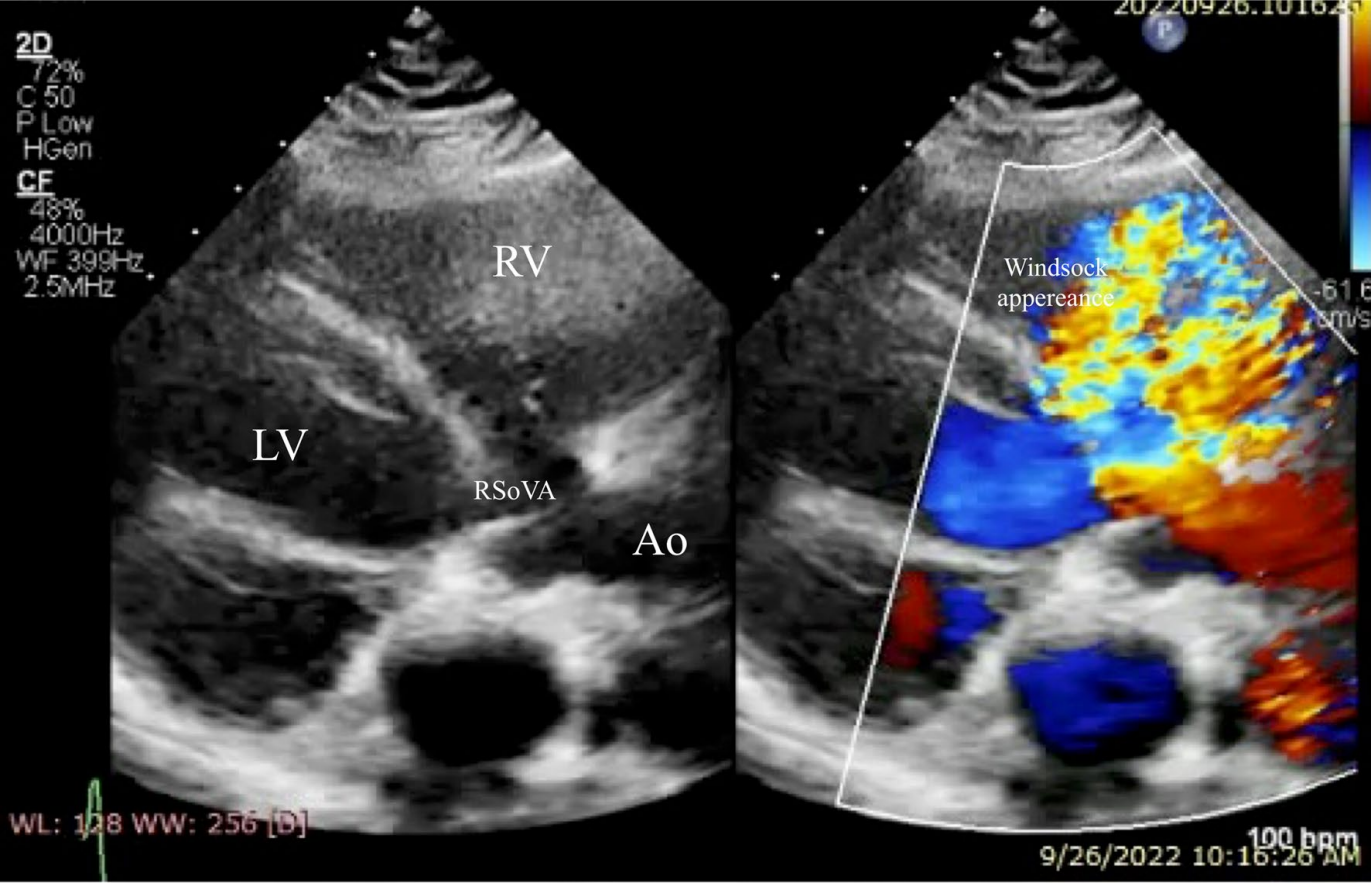
Transthoracic echocardiography identified a “windsock” appearance on parasternal long axis (PLAX) view. Ao = aorta; RV = right ventricle; LV = left ventricle; RSoVA: ruptured sinus of Valsalva aneurysm

**Fig. 2:**
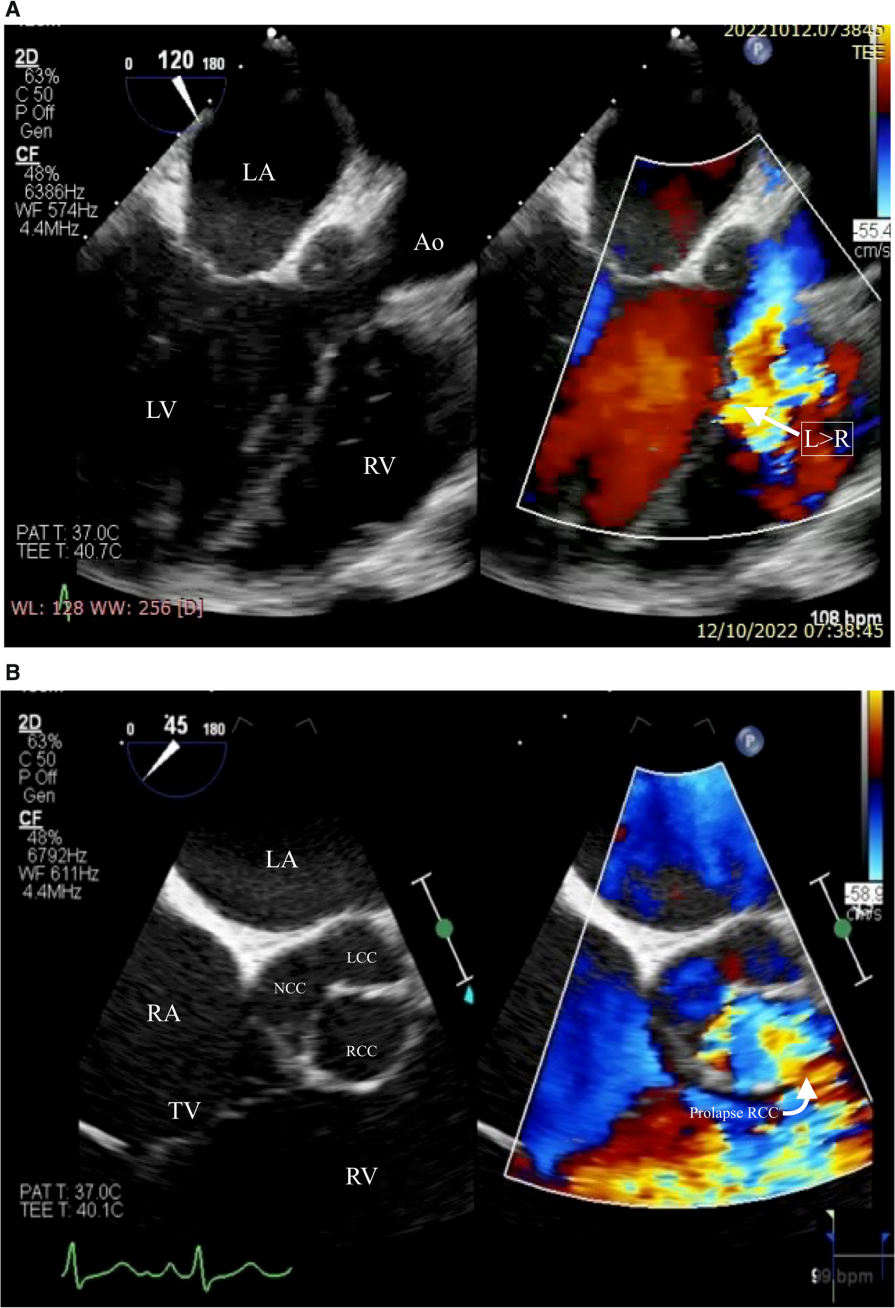
Transoesophageal echocardiography confirmed rupture of the sinus of Valsalva aneurysm on the Right coronary cusps (RCC). **A** and **B** Images taken flow from the RCC to the right ventricle on color doppler (arrow). Ao = aorta; RV = Right ventricle; LV = left ventricle; LA = left atrium; L = left; R = right; TV = tricuspid valve; NCC = non-coronary cusp; LCC = left coronary cusp; RCC = right coronary cusp

Turbulent Doppler flow analysis unveiled the presence of an aneurysmal dilation within the right sinus of Valsalva, projecting into the RV, as depicted in Fig. 1. These findings were subsequently corroborated by transesophageal echocardiography (TEE), which confirmed the presence of a RSoVA extending into the RV. Additionally, TEE raised suspicion of severe pulmonary hypertension, as illustrated in Fig. 2. TEE revealed the presence of a RSoVA characterized by left-to-right shunting. The aneurysm was measured to be 14 mm in diameter and exhibited multiple end holes. Importantly, TEE suggested a high likelihood of severe pulmonary hypertension, while both left and right ventricular functions were noted to be preserved during the assessment.

Preceding the surgical intervention, the patient underwent a comprehensive assessment through right and left heart catheterization procedures. These examinations unveiled the presence of a RSoVA that formed an aorta-to-RV fistula, characterized by multiple end-holes. Importantly, the observed fistula exhibited high flow with low resistance. The pulmonary flow to systemic flow ratio (QP/QS) was determined to be 2.5, and the pulmonary arteriolar resistance index was measured at 1.08 WU m^2^. The pulmonary arteriolar resistance itself was quantified as 0.01 WU, and the pulmonary vascular resistance (PVR) to systemic vascular resistance (SVR) ratio stood at 0.02. Intriguingly, the imaging studies conducted during these catheterization procedures did not reveal any evidence of a VSD flow, as demonstrated in Fig. 2. The definitive diagnosis for this patient encompasses the presence of a RSoVA forming an aorta-to-RV fistula, characterized by the existence of multiple end holes. In addition, there exists a suspicion of a small VSD, which may be obscured by the prolapsed RCC. This potential coverage of the VSD by the prolapsed RCC shows the absence of a discernible shunt on TTE and TEE during right and left heart catheterization. In light of this diagnosis, the patient was initially scheduled for an urgent surgical intervention, specifically surgical repair of the RSoVA. However, the patient and her family, apprehensive about potential side effects associated with surgical management, opted against proceeding with the recommended surgical procedure. Subsequently, the patient was regrettably lost to follow-up while undergoing oral medication therapy.

### Case II

**Fig. 3:**
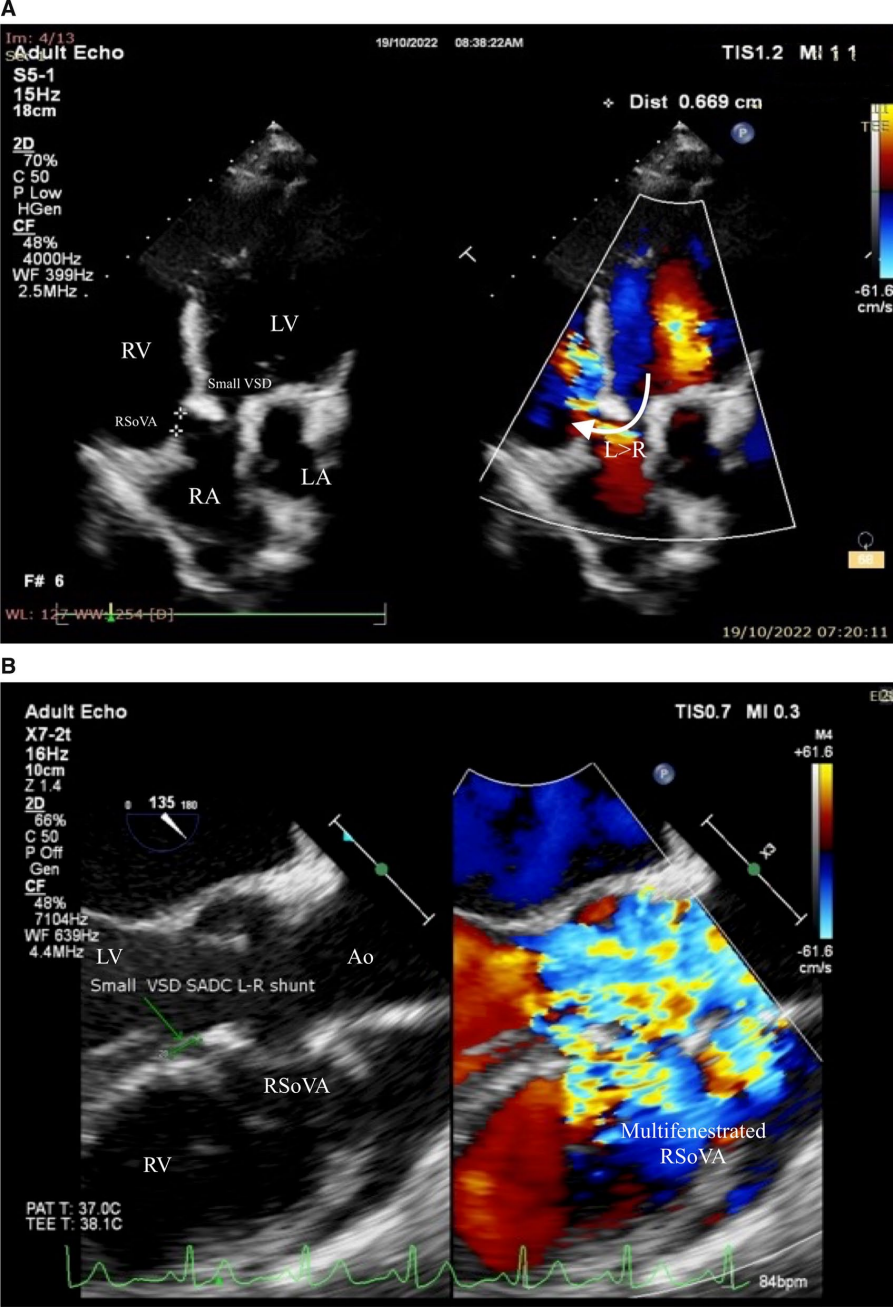
Transthoracic echocardiography presented **A** a small VSD sub aortic ventricular septal defect with a left-to-right shunting (arrow), viewed from an apical 5 chamber view; **B** ME PLAX view on transoesophageal echocardiography. Ao = Aorta; RV = Right ventricle; LV = Left ventricle; LA = Left atrium; L = Left; R = Right; RSoVA: Ruptured sinus of Valsalva aneurysm; VSD = Ventricular septal defect; SADC = Sub aterial doubly committed

A 27-year-old female patient presented at our outpatient clinic, reporting symptoms of dyspnea upon exertion. This patient had a longstanding history of palpitations and exertional dyspnea dating back to her childhood. In her earlier years, the patient received a diagnosis of VSD but did not undergo further medical intervention. Upon physical examination, the patient exhibited hemodynamic stability, with oxygen saturation levels measuring 99% under room air conditions. A continuous murmur of grade V/VI intensity was auscultated along the left sternal border, accompanied by a palpable thrill radiating to the surrounding anatomical region. TTE subsequently revealed the presence of a RSoVA that displayed continuous left-to-right shunting, specifically from the aorta to the RV. Furthermore, TTE identified moderate aortic valve regurgitation, prolapse of the RCC, mild subaortic stenosis, mild tricuspid valve regurgitation, along with an intermediate probability of pulmonary hypertension. Mild mitral valve regurgitation was also noted, in conjunction with left atrial and ventricular dilation. Importantly, both left and right ventricular functions were preserved. Colour Doppler imaging additionally demonstrated continuous wave patterns, indicative of high turbulence occurring during both the systolic and diastolic phases of the left ventricle's cardiac cycle, as illustrated in Fig. 3.

**Fig. 4:**
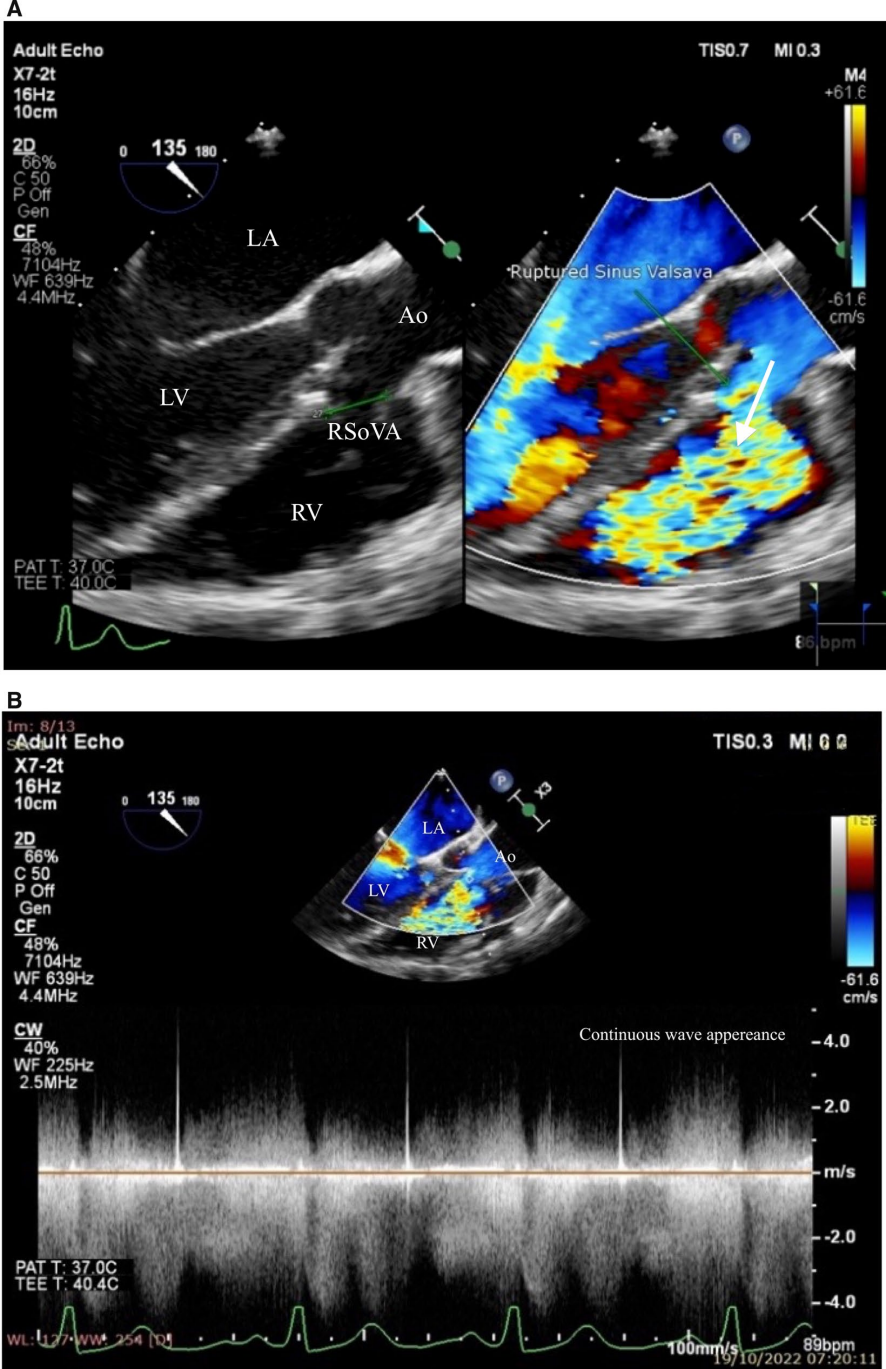
Transoesophageal echocardiography showed **A** a rupture of the Valsalva aneurysm sinus with multiple end- hole with flow heading towards the RV, coming from the aorta to RV (arrow). **B** Continuous wave appearance can be seen CW doppler was done. Ao = Aorta; RV = Right ventricle; LV = left ventricle; LA = left atrium; RSoVA = ruptured sinus of Valsalva aneurysm

**Fig. 5:**
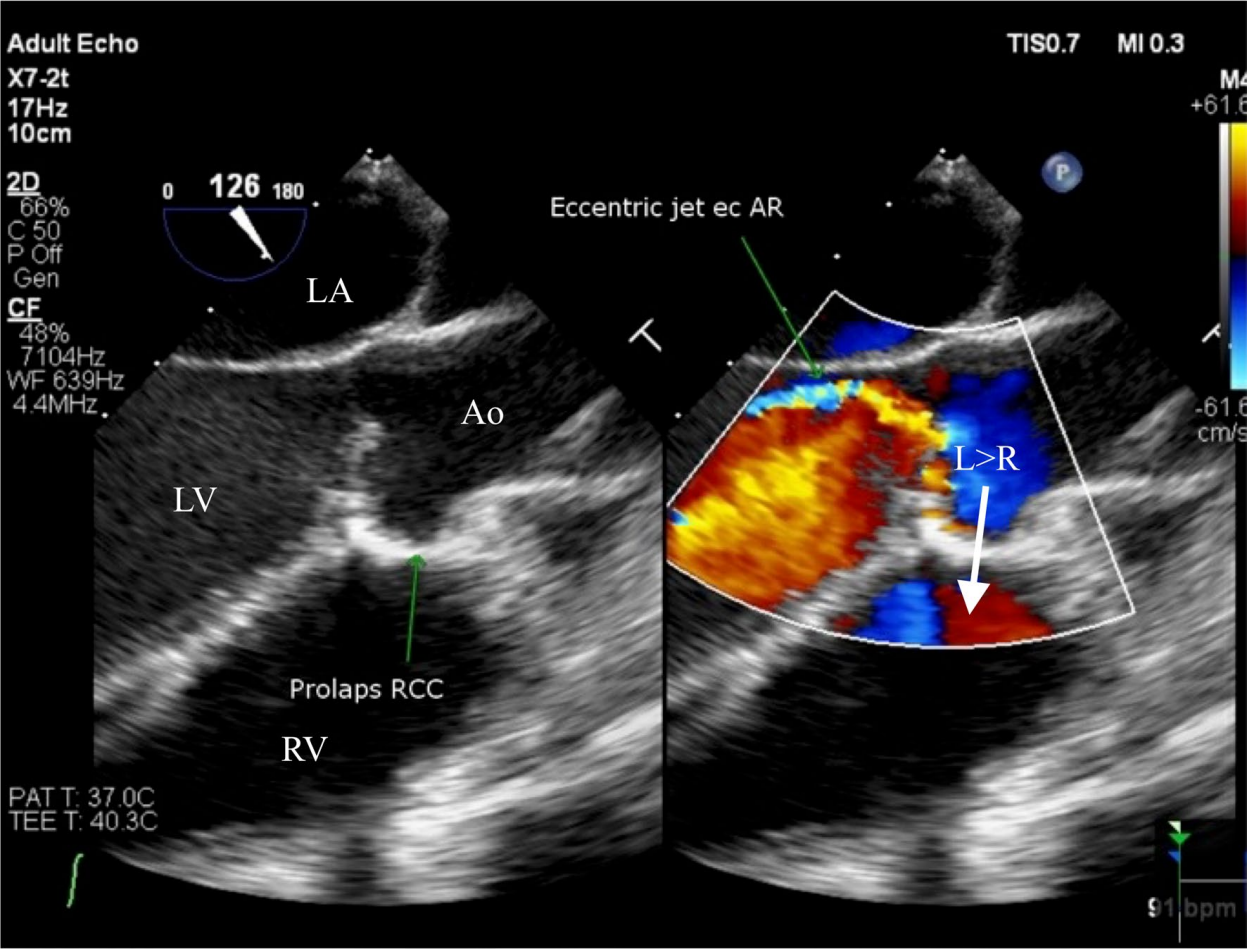
Transoesophageal echocardiography showed RCC Prolapse caused moderate AR. Ao = aorta; RV = right ventricle; LV = left ventricle; LA = left atrium; RCC = right coronary cusp; L = left; r = Right

Additionally, the TEE examination revealed the presence of a small VSD, measuring approximately 4.12 mm in diameter. This VSD was located at the sub arterial doubly committed (SADC) position, exhibiting a left-to-right shunt extending from LV to RV. Notably, the intra-atrial septum appeared to be intact, as depicted in Fig. 4. Furthermore, the examination identified the RSoVA situated within the RCC, with flow directed towards the RV. An eccentric jet resulting from RCC prolapse was responsible for the observed moderate aortic regurgitation, as illustrated in Fig. 5. Consequently, the final diagnosis for this patient encompasses a small VSD with left-to-right shunting, moderate Aortic Regurgitation (AR), mild subaortic stenosis, mild Tricuspid Regurgitation (TR), along with an intermediate probability of pulmonary hypertension. Additionally, mild Mitral Regurgitation (MR) was identified, with both left and right ventricular functions noted as normal. Currently, the patient is scheduled for a right and left heart catheterization procedure. Initially, plans were made for VSD closure through surgical intervention, in combination with surgical repair of the Sinus of Valsalva rupture and aortic valve replacement, all guided by TEE. However, the patient and her family have not yet made a decision regarding surgical intervention, opting instead for oral medication therapy. The prescribed medication regimen includes Ramipril, Bisoprolol, Spironolactone, and Furosemide, with the aim of alleviating symptoms and improving the patient's condition. Regular monthly monitoring of symptoms and echocardiographic examinations is being carried out to assess the patient's progress.

### Case III

A 20-year-old woman has been experiencing progressively worsening dyspnea, classified as New York Heart Association (NYHA) functional class III, and palpitations over the course of the last two years. Her symptoms notably worsened during her second pregnancy, which occurred two years ago. Additionally, the patient frequently encounters angina symptoms during routine activities, often accompanied by pain radiating to her back and episodes of cold sweats. Furthermore, both lower limbs exhibit swelling during strenuous physical activities. There is no medical history of syncope or rheumatic fever in the patient's records. Upon physical examination, the patient presented with stable hemodynamics, with peripheral oxygen saturation measured at 98% while breathing room air. Auscultation of the thorax revealed the presence of a continuous grade V/VI murmur along the left sternal border, accompanied by a palpable thrill that radiated to the surrounding area. These findings indicate the presence of a continuous murmur. The ECG displayed a normal sinus rhythm. TTE provided valuable insights into the patient's cardiac condition. The examination revealed several notable observations, including the dilation of the LA and LV, eccentric hypertrophy of the LV, and normal LV systolic function, with a left ventricular ejection fraction (LVEF) calculated at 66% using the Teichholz method. The assessment also confirmed normal RV contractility, with a tricuspid annular plane systolic excursion (TAPSE) measurement of 33 mm. Other findings included the presence of a tricuspid aortic valve, mild subaortic stenosis with a pressure gradient of 21 mmHg, mild aortic regurgitation accompanied by diastolic flow reversal due to RCC prolapse, mild MR, and mild pulmonary regurgitation (PR) attributed to the prolapsed pulmonary valve. Moreover, a 5 mm VSD was detected, characterized by a left-to-right shunt. To further evaluate the morphology of the aortic and pulmonary valves, TEE was performed as part of the diagnostic process.

**Fig. 6:**
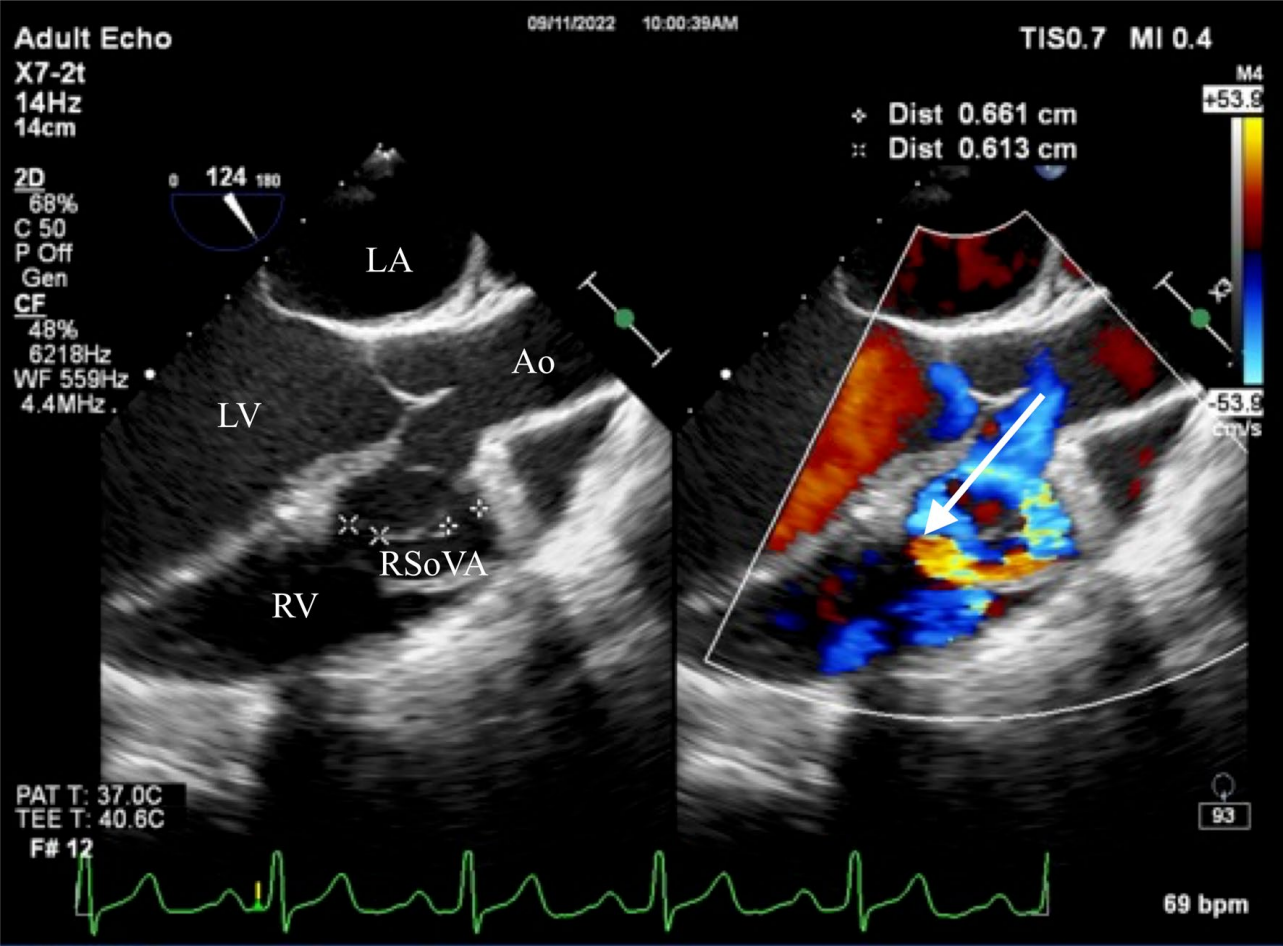
Transoesophageal echocardiography showed rupture of the sinus of Valsalva aneurysm in the RCC region (diameter 1.5 cm) with multi fenestrated (diameter 0.61 to 0.66 cm) towards RV (arrow). Ao = aorta; RV = right ventricle; LV = left ventricle; LA = left atrium; RSoVA = ruptured sinus of Valsalva aneurysm

**Fig. 7:**
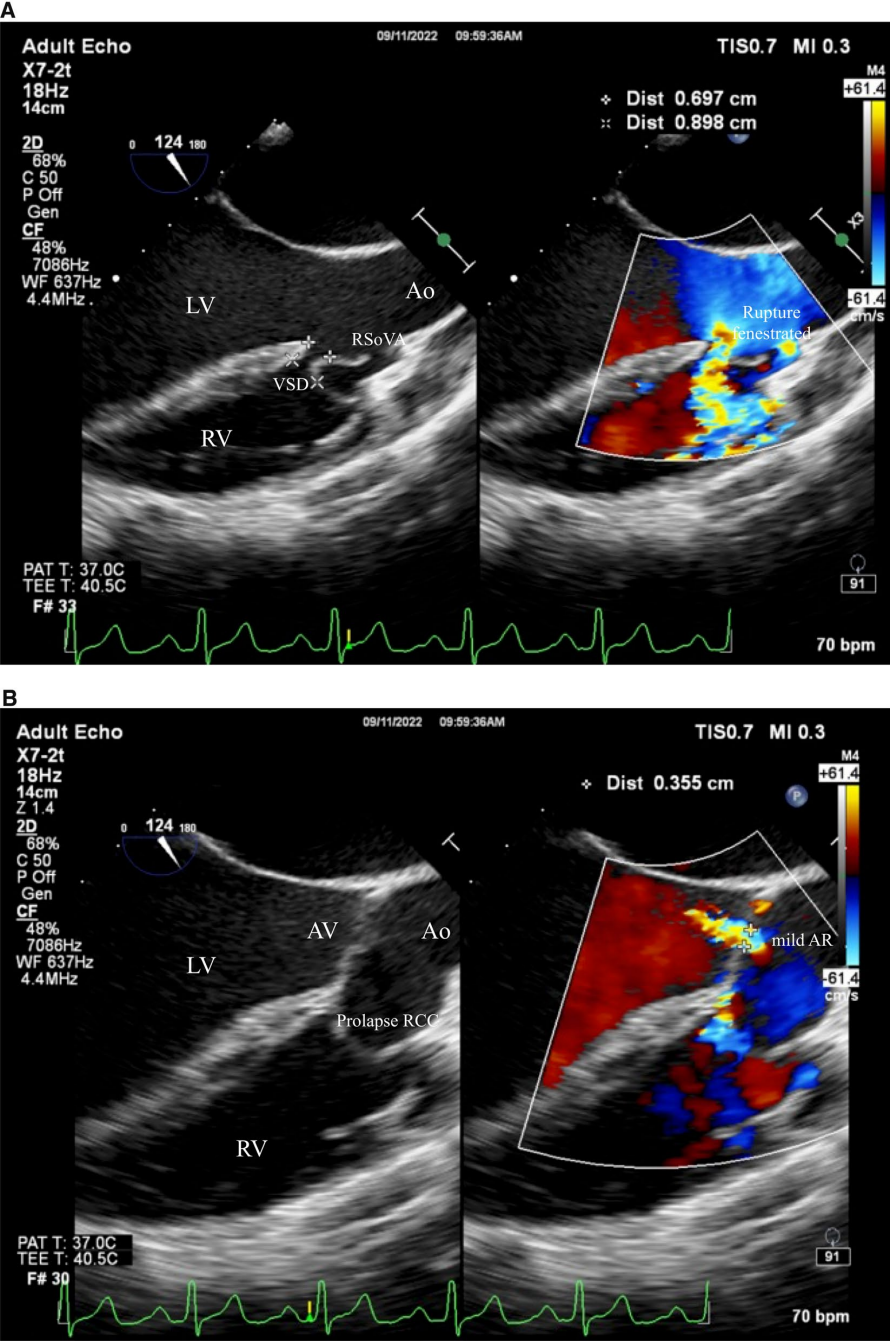
**A** Transoesophageal echocardiography showed subaortic VSD left-to-right shunt. **B** Mild aortic regurgitation due to prolapse of RCC. Ao = aorta; RV = right ventricle; LV = left ventricle; RSoVA = ruptured sinus of Valsalva aneurysm; VSD = Ventricular septal defect

During the examination, a small VSD was identified with a measured diameter ranging between 6.9 and 8.9 mm. This VSD was located in the subaortic region and was associated with a left-to-right shunt. The intra-atrial septum exhibited no apparent defects, but multiple end holes were observed (Fig. 6). The Mid-esophageal Long-Axis (MELAX) view provided a clear visualization of a RSoVA situated within the region of the RCC. This RSoVA had a diameter of 1.5 cm and featured multi-fenestrated openings, each measuring between 0.61 and 0.66 cm in diameter. Importantly, these fenestrations directed the flow towards the right ventricle, resulting in a left-to-right shunt (Fig. 7). Additionally, the presence of an eccentric jet and RCC prolapse was noted, contributing to mild AR. Parameters indicative of AR severity included a pressure half-time (PHT) of 409 ms, an effective regurgitant orifice area (ERO) of 0.12 cm^2^, and a regurgitant volume (RegVol) of 36 ml. Mild subaortic stenosis caused by RCC prolapse was also documented, characterized by an aortic valve maximum velocity (AV Vmax) of 2.35 m/s and a peak gradient (AV Max PG) of 22 mmHg. Furthermore, mild TR was detected, with an intermediate probability of concurrent pulmonary hypertension. Moderate MR was also present. Notably, the examination revealed LA and LV dilatation, yet maintained normal LV systolic function, as demonstrated by a LVEF calculated using the biplane method (BP) at 60.4%. RV function remained within the normal range, with a TAPSE measurement of 32 mm and global normokinetic motion. Based on these findings, the final diagnosis for this patient is Congestive Heart Failure classified as Functional Class II, complicated by adult CHD and a small subaortic VSD featuring a left-to-right shunt from LV to RV. Additionally, the diagnosis encompasses RSoVA with multi-fenestrated left-to-right shunting, mild AR attributable to RCC prolapse, mild PR, and moderate MR. The patient's treatment plan included surgical VSD repair in conjunction with sinus Valsalva rupture repair and aortic valve replacement. To alleviate symptoms, the patient was prescribed an oral medication regimen consisting of Ramipril, Bisoprolol, Nitrate, Spironolactone, and Furosemide. Follow-up appointments were scheduled on a monthly basis to monitor symptom progression and conduct regular echocardiography examinations.

## Discussions

RSoVA displays varying prevalence between Asian (1.2–4.94%) and Western populations (0.5–1.5%) [5]. While non-ruptured SoVA are typically asymptomatic, they can lead to right ventricular outflow tract obstruction and myocardial ischemia due to compression [6]. RSoVA ruptures often coincide with congenital defects, notably VSD, AR, and Bicuspid Aortic Valve (BAV). VSD is more common in Asian populations, while BAV is prevalent in Western populations. Aortic regurgitation incidence is similar in both groups (VSD: Asian 52.4%, Western 37.5%; AR: Asian 33.6%, Western 32.7%; BAV: Asian 0.6%, Western 7.8%) [7]. In Asian countries, most cases of RSoVA originate from the RCC, and 72.5% ruptured into the RV [5]. Our cases align with this pattern, as all three RSoVA cases in our study had RCC origins and RV communication.

In scenarios involving a small VSD situated in the peri-membranous region, the “Venturi effect” becomes pertinent. This phenomenon describes the reduction in fluid pressure as it traverses a restricted or narrow opening. Consequently, pressure transitions from a high-pressure zone to a lower-pressure one, leading to an acceleration in flow velocity from the LV into the RV via the peri-membranous VSD orifice. This alteration in pressure imparts a force that influences the movement of the aortic cusps or sac, drawing them in the direction of the flow or towards the RV [8]. The aortic wall primarily comprises collagen and elastin. With age, the ascending aorta stiffens due to increased collagen. The aortic root has elastin and collagen in its middle layer, while the sinus layer is similar to the ascending aorta. Collagen types I and III make up 80–90% of aortic collagen. The ascending aorta has denser elastin and irregular thickness, whereas the Valsalva sinus has thinner walls with more elastin. Regular collagen distribution is more evident in the ascending aorta than the Valsalva sinus [9]. CHD like supra-crystal VSD or small membranous septal defects are closely associated with the occurrence of SoVA [10]. This linkage is due to increased flow velocity resulting from a decrease in pressure within larger spaces, thereby heightening the risk of SoVA rupture. The “wind sock” appearance, often observed in echocardiography, is attributed to the “Bernoulli effect” [8].

The TTE examination in patients with RSoVA employs 2D echocardiography to assess the dimensions of cardiac chambers. 2D color Doppler is employed to visualize the morphology of the Sinus of Valsalva, ascertain the flow through the rupture, and analyze concomitant congenital anomalies like VSD and valve regurgitation. The optimal visualization of the SoVA is achieved through parasternal long-axis (PLAX) and short-axis (PSAX) views, specifically at the level of the aortic root. Pulsed Wave Doppler (PW) and Color Wave Doppler (CW) findings offer precise localization of the shunt [11, 12]. A characteristic feature of SoVA is the appearance of a “windsock,” signifying the dilation of the Sinus of Valsalva and this characteristic was presence in all three patients [13]. Doppler assessments reveal continuous high-velocity flow from the aorta into the cardiac chambers. The definitive diagnosis typically relies on color Doppler flow mapping. Color flow mapping, complemented by PW Doppler and CW Doppler, elucidates the continuous mosaic flow jet originating from the aneurysm and entering the cardiac chambers [14]. A more detailed examination of the short axis of the left ventricular outflow tract and aortic root aids in distinguishing ventricular septal defect aneurysms from SoVA situated above the aortic annulus. Additionally, PSAX views can facilitate the differentiation of SoVA from coronary arteriovenous fistulas, where dilated coronary arteries may be observed proximate to the aorta [11]. In the presentation of our cases, PW and CW mapping demonstrated continuous waveforms when observed at the PSAX corresponding to the aortic level. Importantly, while the first case showed no signs of valve regurgitation, the second and third cases presented with mild to moderate AR [2].

TEE is particularly useful in assessing concurrent congenital lesions and the rupture of the SoVA. However, it is important to note that TEE does not supersede TTE or angiography in the evaluation of aortic insufficiency or left-to-right shunting [4]. In the first case, initial TTE was performed, and 2D color imaging at the PSAX at the aorta level led to confusion regarding the source of the color flow. Subsequently, TEE examination was conducted to validate the morphological aspects of the aortic valve, SoVA, and the potential presence of coexisting congenital anomalies, such as VSD. Additional TEE examination was undertaken to corroborate the morphology and mechanism of AR and assess the characteristics of RASV. It is noteworthy that TTE and TEE revealed distinct types of VSD: TTE indicated a peri-membranous VSD, while TEE identified a VSD at the SADC location [13]. In the third case, TTE revealed a multi-fenestrated RSoVA that drained into the right ventricle.

In two of the three cases, right and left heart catheterization showed RSoVA forming an aorta to RV fistula with multiple end holes, high flow, low resistance, and multi-fenestrated left-to-right shunting. These findings indicated increased pulmonary artery flow with low pulmonary arteriolar resistance index and a positive oxygen test, suggesting the need for transcatheter closure or surgery. RSoVA can result in aortic valve insufficiency, right ventricular outflow tract obstruction, and myocardial ischemia. Timely surgical intervention is imperative to address these complications once the diagnosis is confirmed [5]. The prognosis following surgical repair of SoVA is generally favorable. Early diagnosis and prompt surgical intervention are essential for enhancing survival rates [15]. The reported preoperative mortality rate stands at 7%, with a long-term survival rate of approximately 63% in extensive case series [7].

While this study offers strengths in presenting three case reports with similar clinical presentations and diagnoses, several limitations warrant attention. Firstly, all three patients did not undergo surgical treatment; instead, they received symptom management and oral medication therapy with monthly outpatient clinic follow-ups. Consequently, this case series cannot evaluate the outcomes or determine the post-surgical prognosis of these patients. Secondly, one out of the three cases was lost to follow-up during oral medication therapy, preventing ongoing monitoring and assessment.

This case report highlights the coexistence of CHD, specifically VSD, with RSoVA, a rare occurrence in the general population. Echocardiography plays a crucial role in its diagnosis, which can be definitively confirmed through right and left heart catheterization procedures. Early diagnosis and urgent surgical repair were recommended in this case series, despite surgical intervention not having been performed as of yet.

## Conclusions

RSoVA is an uncommon but suspected condition in patients with a long history of VSD and a sudden onset of heart failure, characterized by a continuous murmur with a wide pulse pressure. The most common location for RSoVA is the RCC, as observed in all three cases presented here. Two out of the three cases had peri-membranous and subaortic VSD, with subaortic VSD being more frequently reported in cases of sinus of Valsalva aneurysm. Accurate diagnosis and documentation of RSoVA and its relationship with various cardiac chambers can be achieved effectively using 2D color Doppler and pulsed wave Doppler echocardiography. Although RSoVA can lead to significant morbidity, establishing the diagnosis requires appropriate cardiac imaging. Moreover, it is essential to identify other anomalies, especially CHD, which is often missed during clinical examinations. RSoVA is a condition that necessitates urgent surgical intervention, as it carries a high mortality rate if left untreated. Surgical repair remains the preferred and most effective treatment method for RSoVA.

## Data Availability

All data on this case report are included in the published article.

## Abbreviations

AR: Aortic regurgitation
BAV: Bicuspid aortic valve
CHD: Congenital heart disease
CW: Color wave
SoVA: Sinus of Valsalva aneurysm
RSoVA: Ruptured sinus of Valsalva aneurysm
LCC: Left coronary cusp
LA: Left atrium
LV: Left ventricle
MR: Mitral regurgitation
ME LAX: Mid esophageal long axis
NCC: Non-coronary cusp
PR: Pulmonary regurgitation
PLAX: Parasternal long axis
PW: Pulsedwave
PSAX: Parasternal short axis
RCC: Right coronary cusp
RV: Right ventricle
RA: Right atrium
SADC: Sub arterial doubly committed
TR: Tricuspid regurgitation
TEE: Transoesophageal echocardiography
TTE: Transthoracic echocardiography
VSD: Ventricular septal defect
